# Algorithm for Correcting the Keratometric Error in the Estimation of the Corneal Power in Keratoconus Eyes after Accelerated Corneal Collagen Crosslinking

**DOI:** 10.1155/2017/8529489

**Published:** 2017-10-22

**Authors:** David P. Piñero, Vicente J. Camps, Esteban Caravaca-Arens, Dolores de Fez, Francisco J. Blanes-Mompó

**Affiliations:** ^1^Grupo de Óptica y Percepción Visual (GOPV), Department of Optics, Pharmacology and Anatomy, University of Alicante, Alicante, Spain; ^2^Department of Ophthalmology (OFTALMAR), Medimar International Hospital, Alicante, Spain; ^3^Fundación para la Calidad Visual (FUNCAVIS), Alicante, Spain

## Abstract

**Purpose:**

To analyze the errors associated to corneal power calculation using the keratometric approach in keratoconus eyes after accelerated corneal collagen crosslinking (CXL) surgery and to obtain a model for the estimation of an adjusted corneal refractive index (*n*_*k*_adj__) minimizing such errors.

**Methods:**

Potential differences (Δ*P*_c_) among keratometric (*P*_*k*_) and Gaussian corneal power (*P*_c_^Gauss^) were simulated. Three algorithms based on the use of *n*_*k*_adj__ for the estimation of an adjusted keratometric corneal power (*P*_*k*_adj__) were developed. The agreement between *P*_*k*(1.3375)_ (keratometric power using the keratometric index of 1.3375), *P*_c_^Gauss^, and *P*_*k*adj_ was evaluated. The validity of the algorithm developed was investigated in 21 keratoconus eyes undergoing accelerated CXL.

**Results:**

*P*_*k*(1.3375)_ overestimated corneal power between 0.3 and 3.2 D in theoretical simulations and between 0.8 and 2.9 D in the clinical study (Δ*P*_c_). Three linear equations were defined for *n*_*k*_adj__ to be used for different ranges of *r*_1c_. In the clinical study, differences between *P*_*k*_adj__ and *P*_c_^Gauss^ did not exceed ±0.8 D *n*_*k*_ = 1.3375. No statistically significant differences were found between *P*_*k*_adj__ and *P*_c_^Gauss^ (*p* > 0.05) and *P*_*k*(1.3375)_ and *P*_*k*_adj__ (*p* < 0.001).

**Conclusions:**

The use of the keratometric approach in keratoconus eyes after accelerated CXL can lead to significant clinical errors. These errors can be minimized with an adjusted keratometric approach.

## 1. Introduction

Our research group has recently published a series of articles reporting the differences obtained theoretically and clinically between the central corneal power estimated using the classical keratometric approach (keratometric corneal power, *P*_*k*_) and that obtained using the Gaussian equation that considers the curvature of both corneal surfaces and corneal thickness (Gaussian corneal power, *P*_c_^Gauss^) in healthy [1, 2] and postmyopic LASIK corneas [3]. In the healthy cornea, *P*_*k*(1.3375)_ can theoretically overestimate the corneal power (considering *P*_c_^Gauss^ as the reference) up to 2.50 D and in post-LASIK eyes up to 3.50 D if a keratometric corneal refractive index (*n*_*k*_) of 1.3375 is used. A variable keratometric corneal refractive index depending on *r*_1c_ (adjusted keratometric index, *n*_*k*_adj__) was proposed and clinically validated by our research group as an approach to minimize the error associated to the keratometric estimation of corneal power in healthy and post-LASIK eyes [1–3].

In keratoconus eyes, theoretical and clinical errors associated to the calculation of central corneal power considering *P*_*k*_ have been also analyzed and compared with *P*_c_^Gauss^ [4]. In theoretical simulations, an overestimation of *P*_*k*(1.3375)_ was observed in most of cases, with differences among Gaussian and keratometric calculations (Δ*P*_c_ = *P*_*k*(1.3375)_ − *P*_c_^Gauss^) ranging from −0.1 to 4.3 D, depending on the *r*_1c_/*r*_2c_ combination and the theoretical eye model considered. Clinically, *P*_*k*(1.3375)_ was found to overestimate corneal power in a range between 0.5 and 2.5 D, with a mean clinical difference (Δ*P*_c_) of 1.48 D [4, 5]. The clinical value of the keratometric corneal refractive index matching *P*_*k*_ and *P*_c_^Gauss^(*n*_*k*_exact__) ranged from 1.3225 to 1.3314 in a keratoconus population evaluated in a previous study [4]. Eight linear algorithms were developed to estimate the most adequate adjusted corneal refractive index (*n*_*k*_adj__) minimizing the error associated to the corneal power calculation using the keratometric approach in keratoconus. The value of *n*_*k*_adj__ ranged from 1.3190 to 1.3324, and from 1.3207 to 1.3339 using the Gullstrand and Le Grand eye models, respectively. Using this *n*_*k*_adj__for corneal power calculation, differences between *P*_*k*_adj__ and *P*_c_^Gauss^ were found to be clinically in the range within ±0.70 D. The aim of the current study was to analyze theoretically and clinically the errors associated to corneal power calculation using the keratometric approach in keratoconus eyes after accelerated corneal collagen crosslinking surgery and to obtain a model for the estimation of an adjusted corneal refractive index (*n*_*k*_adj__) minimizing such errors.

## 2. Methods

### 2.1. Theoretical Calculations

Central corneal power was calculated using the classical keratometric corneal refractive index (1) and also using the Gaussian equation (2) that considers the contribution of both corneal surfaces and corneal thickness. Differences among both types of central corneal power calculations were determined (4 and 6) and modelled by regression analysis. All calculations and simulations were performed using the Matlab software (Math Works Inc., Natick, MA, USA).

### 2.2. Calculation of the Gaussian and Keratometric Corneal Power

The keratometric power (*P*_*k*_) was estimated by means of the following expression:

(1) $$\begin{array}{c} P_{k}=\frac{n_{k}-1}{r_{1c}}, \end{array}$$

where *n*_*k*_ is the keratometric corneal refractive index and *r*_1c_ is the radius of the anterior corneal surface.

The Gaussian corneal power was calculated by using the formula based on Gaussian optics in paraxial approximation:

(2) $$\begin{array}{c} P_{c}^{Gauss}=P_{1c}+P_{2c}-\delta P_{1c}P_{2c}=\frac{n_{c}-n_{a}}{r_{1c}}+\frac{n_{ha}-n_{c}}{r_{2c}}-\frac{e_{c}}{n_{c}}\frac{n_{c}-n_{a}}{n_{c}}\frac{n_{ha}-n_{c}}{r_{2c}}, \end{array}$$

where *P*_c_^Gauss^ is the total Gaussian corneal power, *P*_1c_ is the anterior corneal power, *P*_2c_ is the posterior corneal power, *r*_1c_ is the anterior corneal radius, *r*_2c_ the posterior corneal radius, *n*_a_ the refractive index of air, *n*_c_ the refractive index of the cornea, *n*_ha_ the refractive index of the aqueous humor, and *e*_c_ is the central corneal thickness.

### 2.3. Calculation of the Adjusted Corneal Refractive Index

As in our previous studies [1–3, 5], the adjusted corneal refractive index (*n*_*k*_adj__) was defined as the value associated to an equivalent difference in the magnitude of Δ*P*_c_ for the extreme values of *r*_2c_ corresponding to each *r*_1c_ value and eye model. Specifically, for each *r*_1c_ value considered, *n*_*k*_adj__ was obtained with the following equation: Δ*P*_c_(*r*_2c_min__) = Δ*P*_c_(*r*_2c_max__). The adjusted keratometric corneal power (*P*_*k*_adj__) can be calculated using the classical keratometric corneal power formula as follows:

(3) $$\begin{array}{c} P_{k_{adj}}=\frac{n_{k_{adj}}-1}{r_{1c}}. \end{array}$$

### 2.4. Differences among Gaussian and Keratometric Approaches

By using (1) and (2), the differences between the keratometric and the Gaussian corneal power (Δ*P*_c_) were calculated with the following expression:

(4) $$\begin{array}{c} ∆P_{c}=P_{k}-P_{c}^{Gauss}=\frac{n_{k}-1}{r_{1c}}-\left(\frac{n_{c}-n_{a}}{r_{1c}}+\frac{n_{ha}-n_{c}}{r_{2c}}-\frac{e_{c}}{n_{c}}\cdot \frac{n_{c}-n_{a}}{r_{1c}}\cdot \frac{n_{ha}-n_{c}}{r_{2c}}\right). \end{array}$$

Expression (4) was simplified by including the concept of *k* ratio (5) as follows:

(5) $$\begin{array}{c} k=\frac{r_{1c}}{r_{2c}}, \end{array}$$

(6) $$\begin{array}{c} ∆P_{c}=P_{k}-P_{c}^{Gauss}=\frac{n_{k}-1}{r_{1c}}-\left(\frac{n_{c}-n_{a}}{r_{1c}}+\frac{n_{ha}-n_{c}}{\left(\left(r_{1c}\right)/k\right)}-\frac{e_{c}}{n_{c}}\cdot \frac{n_{c}-n_{ha}}{r_{1c}}\cdot \frac{n_{ha}-n_{c}}{\left(\left(r_{1c}\right)/k\right)}\right). \end{array}$$

### 2.5. Calculation of the Exact Keratometric Corneal Refractive Index

The calculation of the exact keratometric corneal refractive index (*n*_*k*_exact__) was performed by making (4) or (6) equal to zero. Considering this, the following expressions were obtained:

(7) $$\begin{array}{c} n_{k_{exact}}=\frac{-e_{c}n_{c}+e_{c}n_{c}^{2}+e_{c}n_{ha}-e_{c}n_{c}n_{ha}-n_{c}^{2}r_{1c}+n_{c}^{2}r_{2c}+n_{c}n_{ha}r_{1c}}{n_{c}r_{2c}} \end{array}$$

or

(8) $$\begin{array}{c} n_{k_{exact}}=\frac{-e_{c}{kn}_{c}+e_{c}{kn}_{c}^{2}+e_{c}{kn}_{ha}-e_{c}kn_{c}n_{ha}+n_{c}^{2}r_{1c}-{kn}_{c}^{2}r_{1c}+kn_{c}n_{ha}r_{1c}}{n_{c}r_{1c}}. \end{array}$$

### 2.6. Determination of the Range of Corneal Curvature in Keratoconus Eyes after Corneal Collagen Crosslinking

For our simulations, the range of potential variation of the anterior and posterior corneal curvature in keratoconus after collagen crosslinking surgery (CXL) was defined considering the information reported in previous studies evaluating the outcomes of CXL [6–10]. The definition of the potential values of *r*_2c_ after CXL that could be used in our theoretical simulations was defined according to previous studies reporting changes occurring in such parameter measured using the Scheimpflug imaging technology [11–13]. According to all previous studies revised, the anterior corneal radius (*r*_1c_) was found to range in keratoconus after CXL between 5.6 and 8.5 mm, and the posterior corneal radius (*r*_2c_) between 4.4 and 7.0 mm [6–10]. Accordingly, *k* ratio was found to range between 1.04 and 1.57.

## 3. Clinical Study

### 3.1. Patients and Examination

The prospective study includes a total of 21 eyes of 14 patients aged between 23 and 69 years. All patients belonged to the Corneal and Anterior Segment Unit of the Ophthalmology Department (OFTALMAR) of the Vithas Internacional Medimar Hospital (Alicante, Spain). The study inclusion criterion was the presence of progressive keratoconus: central topographic steepening of more than 1 D with refractive change of more than 0.50 D in the last 6 months. The standard criterion for diagnosing keratoconus was used: corneal topography revealing an asymmetric bowtie pattern with or without skewed axes and at least one keratoconus sign on slit-lamp examination, such as stromal thinning, conical protrusion of the cornea at the apex, Fleischer ring, Vogt striae, or anterior stromal scar [14]. Although it is known that keratoconus progression arrests in the 3rd or 4th decade of life, we detected and included some cases in which progression of the disease was detected in patients older than 40 years old. It should be considered that although uncommon, progression of the disease in patients in the 5th decade of life has been reported in some specific cases [15]. The exclusion criteria were previous eye surgery and the presence of any type of active eye disease. All patients were properly informed about their inclusion and signed an informed consent form. The study complied with the principles of the Declaration of Helsinki and was approved by the hospital ethics committee.

A complete ophthalmological examination was carried out preoperatively, which included measurement of manifest refraction, uncorrected (UDVA) and corrected distance visual acuity (CDVA), Goldmann applanation tonometry, anterior segment slit-lamp examination, corneal topography and aberrometry with the Sirius system (Costruzioni Strumenti Oftalmici, CSO, Florence, Italy), biometry (IOL Master v.4.3, Carl Zeiss Meditec, Jena, Germany), and eye fundus examination. Postoperatively, patients were reviewed at 1 day and 1 month after surgery.

### 3.2. Surgery

All operations were performed by the same expert surgeon (AA) under topical anaesthesia, using the Avedro KXL cross-linking system (Waltham, MA, United States). After separating the eyelids with a blepharostat and applying the anaesthesia, the procedure began with the instillation, every 90 seconds for a total of 4 minutes, of dextran-free hypoosmolar riboflavin drops containing agents to improve the epithelial permeability, including benzalkonium chloride (Paracel, Avedro, Waltham, MA, United States). A benzalkonium chloride-free 0.25% riboflavin solution (VibeX Xtra, Avedro, Waltham, MA, United States) was then instilled at the same rate for 6 minutes. Once these steps had been completed, ultraviolet radiation was applied for 2 minutes and 40 seconds, using a pulsed light protocol (2 seconds on/1 second off). The total energy irradiated was 7.2 J/cm^2^, and the ultraviolet power was 45 mW/cm^2^. After irradiation, the cornea was rinsed with balanced saline solution. As postoperative treatment, the patient was instructed to apply one drop of antibiotic (Tobrex, Alcon Laboratories, Forth Worth, TX, United States) and epithelializing ointment (Oculos Epitelizante, Thea Laboratories, Clermont-Ferrand, France) every 8 hours and to use artificial tears.

### 3.3. Statistical Analysis

Statistical analysis was performed using the software SPSS version 19.0 for Windows (SPSS, Chicago, IL, USA). Normality of all data distributions was first confirmed by means of the Kolmogorov-Smirnov test. Specifically, the unpaired Student *t*-test and Wilcoxon test were used for comparing the two approaches for *P*_c_ calculation in the theoretical study, keratometric and Gaussian. The Bland-Altman analysis [16] was used for evaluating the agreement and interchangeability of the methods used clinically for obtaining the corneal power (*P*_*k*_, *P*_*k*_adj__, and *P*_c_^Gauss^). Pearson correlation coefficient was used to assess the correlation between ∆*P*_c_ and other clinical parameters analyzed. The same level of statistical significance (*p* value < 0.05) was considered in all analyses.

## 4. Results

### 4.1. Theoretical Study

#### 4.1.1. Exact (*n*_*k*_exact__) and Adjusted Keratometric Corneal Refractive Index (*n*_*k*_adj__)

The value of *n*_*k*_exact__ considering all possible combinations of *r*_1c_ (5.6 to 8.5 mm) and *r*_2c_ (4.4 to 7.0 mm) ranged from 1.3140 to 1.3351 for the Gullstrand eye model (Table 1) and from 1.3157 to 1.3366 for the Le Grand eye model (Table 2).

The value of *n*_*k*_adj__ ranged from 1.3210 to 1.3309 and from 1.3227 to 1.3325 for the Gullstrand and Le Grand eye models, respectively (Tables 1 and 2). All *n*_*k*_adj__ values adjusted perfectly to 3 linear equations (*R*^2^ = 1) for each model, and therefore 3 theoretical algorithms only depending on *r*_1c_ were obtained for the calculation of corneal power (Tables 1 and 2).

#### 4.1.2. Differences between *P*_*k*_ and P_c_^Gauss^

If the Gullstrand eye model was used (*n*_*k*_=1.3315), ∆*P*_c_ ranged from an underestimation of −0.7 D (*r*_1c_ = 5.6/*r*_2c_ = 5.4 mm) to an overestimation of 2.4 D (*r*_1c_ = 6.8/*r*_2c_ = 4.4 mm). If the Le Grand eye model was used (*n*_*k*_ = 1.3304), ∆*P*_c_ ranged from −1.2 D to 2.0 D for the same *r*_1c_ and *r*_2c_ values. When the value of *n*_*k*_ = 1.3375 was used, an overestimation was always found, with ∆*P*_c_ ranging from 0.3 D (*r*_1c_ = 7.3/*r*_2c_ = 7.0 mm) to 3.2 D (*r*_1c_ = 6.7 or 6.8/*r*_2c_ = 4.4 mm) for the Gullstrand model and from 0.1 D (*r*_1c_ = 5.6/*r*_2c_ = 5.4 mm or *r*_1c_ = 7.3/*r*_2c_ = 7.0 mm) to an overestimation of 3.0 D (*r*_1c_ = 6.8 or *r*_2c_ = 4.4 mm) for the Le Grand eye model.

#### 4.1.3. Differences between *P*_*k*_adj__ and *P*_c_^Gauss^

*P*_*k*_adj__ ranged from 37.8 D to 59.1 D, whereas *P*_c_^Gauss^ ranged from 36.9 to 59.9 D for the Gullstrand eye model (Table 1). With the Le Grand eye model (Table 2), *P*_*k*_adj__ was found to range between 38.0 and 59.4 D and *P*_c_^Gauss^ between 37.1 and 58.6 D. As shown in Tables 1 and 2, differences between *P*_*k*_adj__ and *P*_c_^Gauss^(Δ*P*_c_) did not exceed the value of ±0.8 D.

### 4.2. Clinical Study

The clinical study comprised 21 eyes of 14 patients with keratoconus, 2 women (14%) and 12 men (86%), with a mean age of 41 ± 17 years (range, 23 to 61 years). The sample comprised 12 (57%) and 9 (43%) left and right eyes, respectively. Main clinical features of the sample evaluated are summarized in Table 3.

#### 4.2.1. Exact (*n*_*k*_exact__) and Adjusted Keratometric Corneal Refractive Index (*n*_*k*_adj__)

The results for *n*_*k*_exact__ and *n*_*k*_adj__ considering the different combinations of *r*_1c_ and *r*_2c_ or *k* values (1.14 to 1.47) are shown in Table 4. The value of *n*_*k*_exact__ ranged from 1.3182 to 1.3312, and the value of *n*_*k*_adj__ ranged from 1.3210 to 1.3306. All these values were also within the range obtained in our previous theoretical simulations (see Table 1).

#### 4.2.2. Agreement of *P*_*k*(1.3375)_ with *P*_c_^Gauss^

An overestimation was always present when *P*_*k*(1.3375)_ was compared with *P*_c_^Gauss^ that ranged between 0.8 and 2.9 D. Statistically significant differences were found between *P*_*k*(1.3375)_ and *P*_c_^Gauss^ (Wilcoxon test, *p* < 0.001). A very strong and statistically significant correlation was found between *P*_*k*(1.3375)_ and *P*_c_^Gauss^ (*r* = 0.99, *p* < 0.001). The Bland-Altman analysis showed a mean difference between *P*_*k*(1.3375)_ and *P*_c_^Gauss^ of 1.63 D, with limits of agreement of 0.44 D and 2.82 D (Table 5).

A very strong statistically significant correlation was found between clinical ∆*P*_c_(*P*_*k*(1.3375)_ − *P*_c_^Gauss^) and *r*_2c_ (*r* = −0.95, *p* < 0.001). The correlation of this ∆*P*_c_ with *r*_1c_, anterior corneal asphericity, and posterior corneal asphericity was moderate (*r*_1c_ = −0.77, *p* < 0.001; QCA = −0.76, *p* < 0.001; and QCP = −0.81, *p* < 0.001), whereas the correlation was weak with the remaining clinical variables evaluated.

#### 4.2.3. Agreement of *P*_*k*_adj__ with *P*_c_^Gauss^

**Figure 1:**
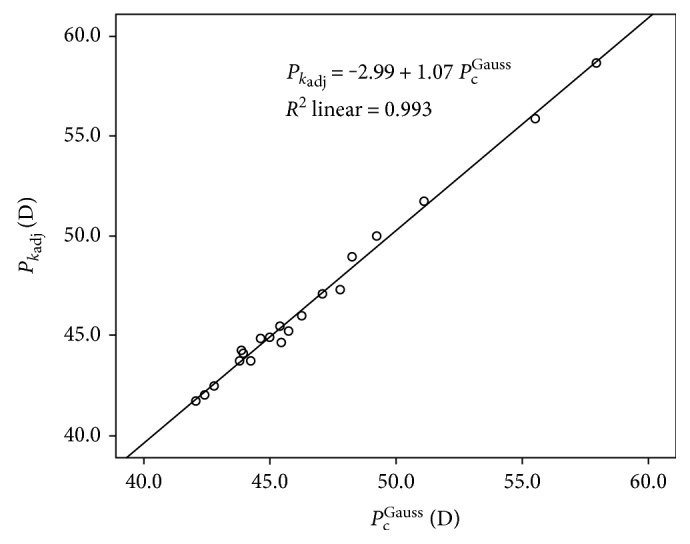
Scatterplot showing the relationship among adjusted keratometric (*P*_*k*_adj__) and Gaussian (*P*_c_^Gauss^) corneal power. The adjusting line to the data obtained by means of the least-squares fit is shown.

**Figure 2:**
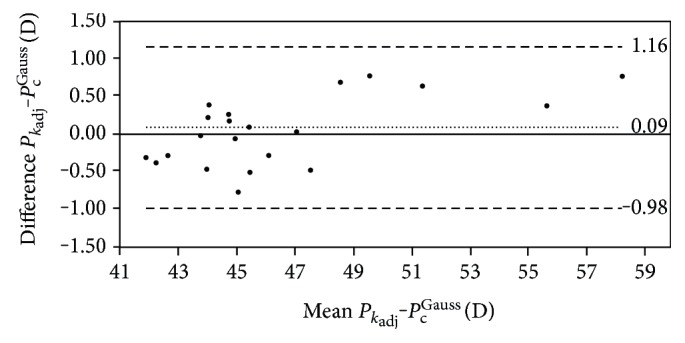
Bland-Altman plot showing the differences between the adjusted keratometric (*P*_*k*_adj__) and Gaussian (*P*_c_^Gauss^) corneal powers against the mean value of both. The upper and lower lines represent the limits of agreement calculated as mean of differences ±1.96 SD.

No statistically significant differences were found between *P*_*k*_adj__ and *P*_c_^Gauss^ (*p* > 0.05), with a very strong and statistically significant correlation between them (*r* = 0.98, *p* < 0.01). A linear dependence was also found between *P*_*k*_adj__ and *P*_c_^Gauss^ (*P*_*k*_adj__ = −2.99 + 1.07 × *P*_c_^Gauss^ , *R*^2^ = 0.99) (Figure 1). According to the Bland and Altman analysis, the range of agreement between *P*_*k*_adj__ and *P*_c_^Gauss^ was 0.09 D, with limits of agreement of −0.98 D and 1.16 D (Figure 2 and Table 5). A moderate correlation of the difference between *P*_*k*_adj__ and *P*_c_^Gauss^(∆*P*_c_) with *r*_2c_ (*r* = −0.66, *p* < 0.01) and the asphericity of the posterior corneal surface was found (*r* = −0.70, *p* < 0.01).

#### 4.2.4. Agreement of *P*_*k*(1.3375)_ with *P*_*k*_adj__

**Figure 3:**
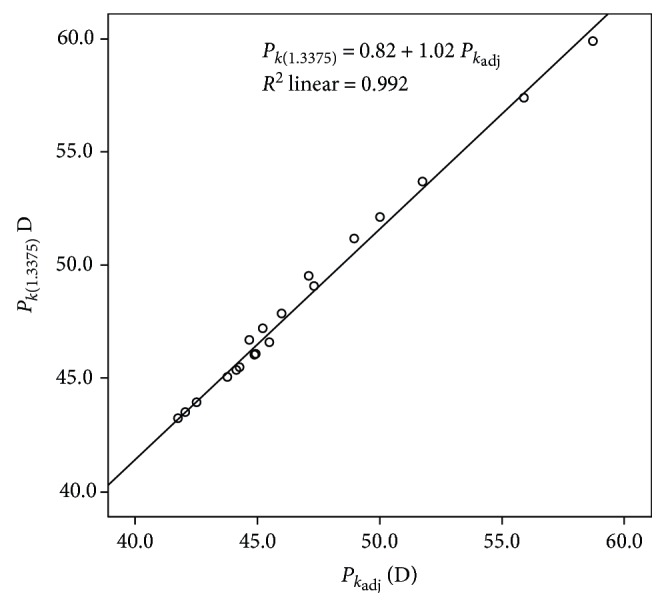
Scatterplot showing the relationship among adjusted keratometric (*P*_*k*_adj__) and classical keratometric (*P*_*k*(1.3375)_) corneal power. The adjusting line to the data obtained by means of the least-squares fit is shown.

**Figure 4:**
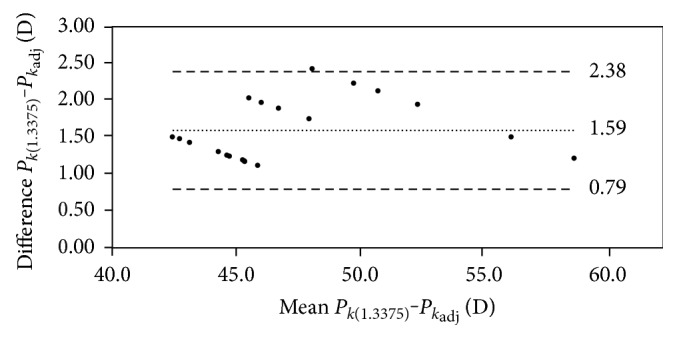
Bland-Altman plot showing the differences between the adjusted keratometric (*P*_*k*_adj__) and classical keratometric (*P*_*k*(1.3375)_) corneal powers against the mean value of both. The upper and lower lines represent the limits of agreement calculated as mean of differences ±1.96 SD.

Statistically significant differences were found between *P*_*k*(1.3375)_ and *P*_*k*_adj__ (*p* < 0.001), with a very strong and statistically significant correlation of such variables (*r* = 0.98, *p* < 0.001) (Figure 3). The Bland-Altman analysis showed a mean difference value between *P*_*k*(1.3375)_ and *P*_*k*_adj__ of 1.59 D, with limits of agreement of 0.79 D and 2.38 D (Figure 4 and Table 5). The value of ∆*P*_c_ between *P*_*k*(1.3375)_ and *P*_*k*_adj__ correlated significantly with *r*_2c_ (*r* = 0.44, *p* < 0.001), *r*_1c_ (*r* = −0.39, *p* < 0.001), and the asphericity of the anterior corneal surface (*r* = −0.43, *p* < 0.001).

## 5. Discussion

Significant differences in corneal power between the keratometric and Gaussian estimations have been observed in our simulation study using the Gullstrand and Le Grand eye models in keratoconus corneas undergoing CXL, which is consistent with the results of previous studies [1–5]. Specifically, the keratometric estimation has been shown to be able to overestimate or underestimate the corneal power depending on *r*_1c_, *n*_*k*_, or the eye model used in normal healthy [1, 2], post-LASIK [3], and keratoconus corneas [4, 5]. In our simulation study, when *n*_*k*_ = 1.3375 was used, *P*_*k*(1.3375)_ overestimated *P*_c_^Gauss^ between +0.30 D and +3.2 D and between +0.1 D and +3.0 D for Gullstrand and Le Grand eye models, respectively. A similar outcome was reported in simulations in nontreated keratoconus corneas, although the maximum potential overestimations were higher (Δ*P*_c_ ranging from −0.08 D to +4.77 D for Gullstrand eye model and from −0.26 D to +3.97 D for Le Grand eye model) [4]. In contrast, the overestimations have been demonstrated to be lower when the classical keratometric approach is used in normal healthy eyes, with maximal potential overestimations of 2.50 and 2.30 D for the Gullstrand and Le Grand eye models, respectively [1]. Likewise, maximal overestimations of 3.55 D and 3.39 D for Gullstrand and Le Grand eye models, respectively, have been obtained in post-LASIK corneas [3]. Therefore, the keratometric approach is an inaccurate procedure to estimate the corneal power, especially in those cases in which the relationship between the anterior and posterior corneal curvature is altered, such as occurrences after laser refractive surgery [3] and in corneal ectatic diseases [17].

The data obtained in our simulations were found to be consistent with those obtained in the clinical study also conducted in the current research. We evaluated a sample of keratoconus corneas undergoing CXL surgery and found that Δ*P*_c_ ranged between +0.8 and +2.9 D when *P*_*k*(1.3375)_ and *P*_c_^Gauss^ were compared. Mean difference between corneal power estimations was +1.6 D, and this difference was statistically significant. A similar outcome was obtained in a previous study evaluating the keratometric error in nontreated keratoconus, with overestimations between +0.7 and +2.4 D and a mean difference between keratometric and Gaussian corneal powers of +1.4 D [5]. Therefore, a small trend to more overestimation of the keratometric approach is observed in keratoconus once a CXL treatment is applied. An explanation for this fact may be the changes occurring with surgery at the posterior corneal surface leading to altered values of the *k* ratio [18]. This overestimation must be considered in clinical practice when the changes in corneal curvature after CXL are analysed in order to avoid overestimating the effect of the surgery.

The corneal refractive index avoiding the error when the keratometric approach is used (*n*_*k*_exact__) was calculated for each *r*_1c_*-r*_2c_ combination in our keratoconus sample with previous CXL. The value of *n*_*k*_exact__ ranged from 1.3140 to 1.3351 for the Gullstrand eye model and from 1.3157 to 1.3366 for Le Grand eye model in our simulations. Clinically, the value of *n*_*k*_exact__ranged from 1.3182 to 1.3312 using the Gullstrand eye model for calculations. This interval is wider than that obtained in nontreated keratoconus eyes, with values ranging from 1.3225 to 1.3314 [5]. This confirms that the variation of *k* ratio in CXL-treated keratoconus eyes is higher due to posterior corneal surface and volumetric changes. Further studies are needed to confirm the real effect on corneal volume of accelerated CXL. As in previous studies evaluating different ocular conditions, the use of the classical keratometric corneal refractive index *n*_*k*_ = 1.3375 was found to be a wrong approach [1, 2, 4, 5].

As devices measuring the curvature of the posterior corneal surface are not available in all clinical settings, an adjusted keratometric approach was developed to calculate the corneal power using the keratometric approximation but with a minimal error associated. We could not use a previous adjusted keratometric algorithm defined by our research group for keratoconus as the variation required for the adjusted corneal refractive index was higher [5]. Consequently, new algorithms were developed using the Gullstrand and Le Grand eye models to obtain the adjusted corneal refractive index (*n*_*k*_adj__) minimizing the error associated to the keratometric corneal power calculation. Specifically, three different algorithms were defined for different ranges of *r*_1c_. With them, *n*_*k*_adj__ was found to range from 1.3210 to 1.3309 for the Gullstrand eye model and from 1.3227 to 1.3325 for the Le Grand eye model. When *P*_*k*_adj__ was compared with *P*_c_^Gauss^ in our theoretical simulations, the differences between both corneal power values did not exceed 0.8 D. This difference of 0.8 D was only observed for the maximum and minimum values of *r*_2c_.

Once the algorithm is developed, we validated it clinically in a sample of 21 keratoconus eyes with previous CXL surgery. In this sample, the value of *n*_*k*_adj__ ranged from 1.3210 to 1.3306, which was consistent with the range found in our theoretical simulations. No statistically significant differences were found between *P*_*k*_adj__ and *P*_c_^Gauss^, with a very strong and statistically significant correlation between both values. The mean difference was +0.09 D, with 85% of cases showing a difference of 0.7 D or below and 76% of cases showing a difference of 0.5 D or below. Therefore, if *r*_2c_ is not available or cannot be measured, the keratometric approach can be used to estimate the corneal power in keratoconus eyes with previous CXL surgery with an acceptable error associated in most of cases. Similar results were obtained in our previous study in nontreated keratoconus corneas using a specific adjusted keratometric algorithm [5]. In such study, no statistically significant differences were also found between *P*_*k*_adj__ and *P*_c_^Gauss^, with a mean difference of +0.04 D. Besides this analysis, we confirmed in the clinical sample that the classical keratometric approach based on the use of the corneal refractive index of 1.3375 provided a very significant overestimation of the corneal power, with a mean difference between *P*_*k* (1.3375)_ and *P*_c_^Gauss^ of +1.63 D. As in healthy corneas [1, 2] as well as in post-LASIK [3] and keratoconus corneas [4, 5], the keratometric value of 1.3375 is not valid for corneal power calculation in keratoconus eyes with previous CXL surgery.

There are some potential weaknesses in this study, such as the use of a limited number of theoretical eye models for the simulations or the use of paraxial optics, not considering the effect of corneal asphericity on Δ*P*_c_. However, the purpose of the study was only to evaluate the error in the estimation of the central corneal power where paraxial optics can be applied without errors, which is the easiest and fastest procedure for the clinical practice. Regarding the clinical study, the sample size was limited and it can be considered as a preliminary study. However, it should be considered that it is the first study evaluating the error associated to keratometric approach for corneal power calculation in keratoconus eyes with previous CXL and the clinical results are completely consistent with those obtained in simulations. Future studies should be done to confirm our results with a larger number of cases as well as to evaluate the real benefit of using our adjusted algorithm for corneal power estimation in intraocular lens power calculation after CXL. Likewise, the potential usefulness of our algorithm in keratoconus eyes undergoing crosslinking using other different techniques (epi-off, iontophoresis) must be investigated.

In conclusion, the use of a single value of *n*_*k*_ for the estimation of the corneal power using the keratometric approach is not valid in eyes with keratoconus and previous CXL surgery and can lead to significant errors. Specifically, the use of the classical keratometric corneal refractive index of 1.3375 to estimate the corneal power using the keratometric assumption must be avoided as it leads to significant levels of overcorrection of corneal power. This can be minimized using a variable adjusted corneal refractive index (*n*_*k*_adj__) if the technology required for the measurement of the posterior corneal curvature is not available. This variable corneal refractive index is dependent on the keratoconus stage. Changes in this algorithm due to post-CXL time should be also investigated in future studies.

## Figures and Tables

**Table 1:** Algorithms for *n*_*k*_exact__ and *n*_*k*_adj__ developed using the Gullstrand eye model for different *r*_1c_ and/or *k* intervals. Likewise, the corresponding theoretical ranges for *n*_*k*_adj__, *P*_*k*_adj__, and *P*_*c*_^Gauss^ and differences (Δ*P*_c_) between *P*_*k*_adj__ and *P*_c_^Gauss^ are also shown. Minimum and maximum *n*_*k*_adj__, *P*_*k*_adj__, and *P*_c_^Gauss^ values are bolded in the table.

*r*_1c_ (mm)	[*k*_min_*,k*_max_]	*n*_*k*_adj__ algorithm	*n*_*k*_adj__	*n*_*k*_exact__	*P*_c_^Gauss^ (D)	*P*_*k*_adj__ (D)	Δ*P*_c_ (D)
[5.6, 6.8]	[1.04, 1.55]	−0.00825 *r*_1c_ + 1.3771	[**1.3210**, **1.3309**]	[1.3154, 1.3355]	[46.4, **59.9**]	[47.2, **59.1**]	[−0.8, 0.8]
[6.9, 7.2]	[1.15, 1.50]	−0.00750 *r*_1c_ + 1.3770	[1.3230, 1.3253]	[1.3171, 1.3309]	[44.0, 48.0]	[44.9, 47.1]	[−0.8, 0.8]
[7.3, 8.5]	[1.04, 1.57]	−0.00656 *r*_1c_ + 1.3769	[1.3211, 1.3290]	[**1.3140**, **1.3351**]	[**36.9**, 45.9]	[**37.8**, 45.1]	[−0.8, 0.8]

**Table 2:** Algorithms for *n*_*k*_exact__ and *n*_*k*_adj__ developed using the Le Grand eye model for different *r*_1c_ and/or *k* intervals. Likewise, the corresponding theoretical ranges for *n*_*k*_adj__, *P*_*k*_adj__, and *P*_c_^Gauss^ and differences (Δ*P*_c_) between *P*_*k*_adj__ and *P*_c_^Gauss^ are also shown. Minimum and maximum *n*_*k*_adj__, *P*_*k*_adj__, and *P*_c_^Gauss^ values are bolded in the table.

*r*_1c_ (mm)	[*k*_min_*,k*_max_]	*n*_*k*_adj__ algorithm	*n*_*k*_adj__	*n*_*k*_exact__	*P*_c_^Gauss^ (D)	*P*_*k*_adj__ (D)	Δ*P*_c_ (D)
[5.6, 6.8]	[1.04, 1.55]	−0.00819 *r*_1c_ + 1.3783	[**1.3227**, **1.3325**]	[1.3171, 1.3370]	[46.6, **58.6**]	[47.4, **59.4**]	[−0.8, 0.8]
[6.9, 7.2]	[1.15, 1.50]	−0.00744 *r*_1c_ + 1.3781	[1.3245, 1.3267]	[1.3188, 1.3324]	[44.3, 48.2]	[45.1, 47.4]	[−0.8, 0.8]
[7.3, 8.5]	[1.04, 1.57]	−0.00651 *r*_1c_ + 1.3781	**[1.3227**, 1.3305]	[**1.3157**, **1.3366**]	[**37.1**, 46.1]	[**38.0**, 45.3]	[−0.8, 0.8]

**Table 3:** Mean ocular features of the clinical sample evaluated in the current study.

Parameter	Mean (SD)	Range
*r*_1c_(mm)	7.1 (0.60)	5.6 to 7.8
*r*_2c_ (mm)	5.6 (0.70)	4.4 to 6.6
*k*	1.2679 (0.09)	1.1404 to 1.4719
*Asphericity anterior surface*	−0.7 (0.53)	−1.6 to 0.3
*Asphericity posterior surface*	−0.8 (0.73)	−2.0 to 0.7
*P*_*k*(1.3375)_(D)	48.2 (4.5)	43.3 to 59.9
*P*_*k*_adj__ (D)	46.6 (4.4)	41.7 to 58.7
*P*_c_^Gauss^ (D)	46.5 (4.1)	42.1 to 57.9
*e*_c_min__ (*μ*m)	452 (47.2)	384 to 546
*e*_c_central__ (*μ*m)	488 (64.6)	418 to 639

**Table 4:** Values of *n*_*k*_exact__ and *n*_*k*_adj__ for different intervals of *r*_1c_ and the difference between them in terms of corneal power (Δ*P*_c_) in the sample of keratoconus eyes undergoing corneal collagen crosslinking evaluated. Minimum and maximum *n*_*k*_exact__ and *n*_*k*_adj__ values are bolded in the table.

*r*_1c_ (mm)	Number of patients	[*k*_min_,*k*_max_]	*n*_*k*_exact__	*n*_*k*_adj__	Δ*P*_c_ (D)
[5.6, 6.8]	6	[1.26, **1.47**]	[**1.3182**, 1.3264]	[**1.3210**, **1.3306**]	[0.0, 0.8]
[6.9, 7.2]	5	[1.20, 1.25]	[1.3261, 1.3287]	[1.3228, 1.3294]	[−0.8, 0.1]
[7.3, 8.5]	10	[**1.14**, 1.30]	[1.3254, **1.3312**]	[1.3257, 1.3289]	[−0.5, 0.4]

**Table 5:** Bland-Altman analysis outcomes of the comparison between different methods of corneal power calculation.

	Δ*P*_c_ ± SD (D)	LoA (D)	*p* value
*P*_*k*(1.3375)_ − *P*_c_^Gauss^	1.63 ± 0.6	0.44 to 2.82	0.000
*P*_*k*(1.3375)_ − *P*_*k*_adj__	1.59 ± 0.4	0.79 to 2.38	0.000
*P*_*k*_adj__ − *P*_c_^Gauss^	0.09 ± 0.5	−0.98 to 1.16	0.794
